# Optimizing Pharmacology Studies in Pregnant and Lactating Women Using Lessons from HIV: A Consensus Statement

**DOI:** 10.1002/cpt.2048

**Published:** 2020-10-15

**Authors:** Ahizechukwu C. Eke, Adeniyi Olagunju, Jeremiah Momper, Martina Penazzato, Elaine J. Abrams, Brookie M. Best, Edmund V. Capparelli, Adrie Bekker, Yodit Belew, Jennifer J. Kiser, Kimberly Struble, Graham Taylor, Catriona Waitt, Mark Mirochnick, Tim R. Cressey, Angela Colbers, on behalf of the participants of the WHO-IMPAACT workshop on “Approaches to Optimize and Accelerate Pharmacokinetic Studies in Pregnant and Lactating Women”

**Affiliations:** 1 Division of Maternal Fetal Medicine, Department of Gynecology & Obstetrics, Johns Hopkins University School of Medicine, Baltimore, Maryland, USA; 2 Faculty of Pharmacy, Obafemi Awolowo University, Ile-Ife, Nigeria; 3 Department of Molecular & Clinical Pharmacology, University of Liverpool, Liverpool, UK; 4 Skaggs School of Pharmacy and Pharmaceutical Sciences, University of California San Diego, La Jolla, California, USA; 5 HIV, Hepatitis and STI Department, World Health Organization, Geneva, Switzerland; 6 Mailman School of Public Health, ICAP at Columbia University, New York, New York, USA; 7 Department of Pediatrics, Vagelos College of Physicians & Surgeons, Columbia University, New York, New York, USA; 8 Pediatrics Department, University of California San Diego School of Medicine-Rady Children’s Hospital San Diego, San Diego, California, USA; 9 University of Liverpool, Liverpool, UK; 10 Department of Paediatrics and Child Health, Stellenbosch University, Cape Town, South Africa; 11 Division of Antiviral Products, US Food and Drug Administration (FDA), Silver Spring, Maryland, USA; 12 Department of Pharmaceutical Sciences, University of Colorado Skaggs School of Pharmacy and Pharmaceutical Sciences, Aurora, Colorado, USA; 13 Department of Infectious Disease, Faculty of Medicine, Imperial College, London, UK; 14 Department of HIV Pharmacology, University of Liverpool, Liverpool, UK; 15 Boston University School of Medicine, Boston, Massachusetts, USA; 16 PHPT/IRD UMI 174, Faculty of Associated Medical Sciences, Chiang Mai University, Chiang Mai, Thailand; 17 Department of Immunology & Infectious Diseases, Harvard T.H. Chan School of Public Health, Boston, Massachusetts, USA; 18 Department of Pharmacy, Radboud Institute for Health Sciences, Radboud University Medical Center, Nijmegen, The Netherlands.

## Abstract

Information on the extent of drug exposure to mothers and infants during pregnancy and lactation normally becomes available years after regulatory approval of a drug. Clinicians face knowledge gaps on drug selection and dosing in pregnancy and infant exposure during breastfeeding. Physiological changes during pregnancy often result in lower drug exposures of antiretrovirals, and in some cases a risk of reduced virologic efficacy. The International Maternal Pediatric Adolescent AIDS Clinical Trials (IMPAACT) network and the World Health Organization (WHO)–convened Pediatric Antiretrovirals Working Group collaboratively organized a workshop of key stakeholders in June 2019 to define key standards to generate pharmacology data for antiretrovirals to be used among pregnant and lactating women; review the antiretroviral product pipeline; describe key gaps for use in low-income and middle-income countries; and identify opportunities to undertake optimal studies allowing for rapid implementation in the clinical field. We discussed ethical and regulatory principles, systemic approaches to obtaining data for pregnancy pharmacokinetic/pharmacodynamic (PK/PD) studies, control groups, optimal sampling times during pregnancy, and pharmacokinetic parameters to be considered as primary end points in pregnancy PK/PD studies. For lactation studies, the type of milk to collect, ascertainment of maternal adherence, and optimal PK methods to estimate exposure were discussed. Participants strongly recommended completion of preclinical reproductive toxicology studies prior to phase III, to allow study protocols to include pregnant women or to allow women who become pregnant after enrolment to continue in the trial. The meeting concluded by developing an algorithm for design and interpretation of results and noted that recruitment of pregnant and lactating women into clinical trials is critical.

## BACKGROUND

Pregnancy is associated with profound physiologic, immunologic, structural, and inflammatory changes.^1^ These dynamic changes occur in the maternal and feto-placental units during pregnancy, and significantly influence the pharmacokinetic (PK) processes of drug absorption,^2^ distribution,^3,4^ metabolism,^1,5^ and excretion.^6^ For women living with HIV, altered pharmacokinetics during pregnancy can often result in lower antiretroviral (ARV) drug exposures,^7–9^ possibly increasing the risk of treatment failure,^10^ maternal HIV disease progression, perinatal transmission, drug resistance,^11^ and maternal death.^11^ Some guidelines recommend dose adaptation of certain ARVs in pregnancy because of lower drug exposures (lopinavir/ritonavir; darunavir/ritonavir).^12^

There is a median gap of 6 years between regulatory approval of an ARV and availability of pregnancy PK data to inform dosing in pregnant women, putting pregnant women and their unborn infants at risk.^13^ A recent example, underscoring the clinical importance of defining the pregnancy-related changes in drug disposition, was evident in the November 2018 revised product labeling of cobicistat-containing ARV regimens.^14^ Despite cobicistat-containing regimens being potent, convenient, and well tolerated, cobicistat exposure and its boosting effect are substantially reduced during pregnancy, resulting in the recommendation that cobicistat-containing fixed-dose combinations should not be used during pregnancy.^14,15^ These revisions were made three to six years after these drugs were first approved for use in nonpregnant adults in the United States. Data during pregnancy are not typically required for drug approval.^13^ The time lag between initial approval of a drug and availability of essential pregnancy-specific PK and safety data are substantial, as pregnancy studies have historically been performed in the postmarketing setting using opportunistic designs (the practice of enrolling pregnant or lactating women who are already taking a prescription medication of interest into a PK study).^8,15^ To shorten this time period, innovative approaches are needed to include pregnant and nonpregnant adults earlier in drug development in a prospective way with standardized methodology during drug development programs.^16^

Given that women living with HIV are breastfeeding their infants, as is recommended in many low-income and middle-income countries (LMIC),^17^ it is important to know the extent of infant exposure to maternal drugs via breastmilk, in order to assess potential consequences. In general, low drug concentrations have been observed in breastmilk of women on ARVs; however, in theory, drug toxicity could occur in infants, for example due to long-term exposure to low levels of ARVs.^18^ Prophylaxis to prevent infection, or a theoretical risk that resistance development from subtherapeutic exposure (e.g., to antimicrobial agents) in infants who acquire infection, can occur. In high-income and some middle-income countries, breastfeeding by women living with HIV is generally not recommended in order to reduce the likelihood of viral transmission to the infant. This prevents opportunistic design lactation studies when new drugs are first approved in these settings. In contrast, in LMIC, mothers living with HIV typically breastfeed for at least 12 months. Yet, ARV transfer into breastmilk and resulting infant ARV drug exposure from breastfeeding remains unknown for several ARVs.^19^

Some progress has been made in addressing the lag in conducting pregnancy and lactation-related safety and PK studies. For example, regulatory bodies including the US Food and Drug Administration (FDA) and the European Medicines Agency (EMA) have recently emphasized the need to facilitate the inclusion of women (pregnant and nonpregnant) in clinical drug development programs.^20–22^ In addition, the FDA and EMA have issued guidance on the conduct of PK and pharmacodynamic (PD) studies in pregnant and lactating women, and have highlighted the importance of leveraging the unique and extensive international ARV registry database generated in pregnant and lactating women living with HIV (PLWHIV) to improve our understanding of medication safety.^23^ Recently, the Task Force on Research Specific to Pregnant Women and Lactating Women (PRGLAC), established by the 21st Century Cures Act, provided a detailed report of research on medication use in pregnancy, and identified 15 recommendations on how to facilitate research and develop safe and effective therapies for pregnant and lactating women.^24^ In addition, a multiyear project funded by the US National Institutes of Health – PHASES (Pregnancy & HIV/AIDS: Seeking Equitable Study), established in 2013, focuses on the ethical issues related to conducting studies in pregnancy and inclusion of pregnant women in clinical trials (http://www.hivpregnancyethics.org/). In 2004, the Obstetric-Fetal Pharmacology Research Centers (OPRC) Network was set up to improve the safety and effective use of therapeutic drugs in women during pregnancy and lactation (https://www.utmb.edu/nichd-oprc/home). Ongoing research activities within the network are focused on the efficacy, pharmacology, placental transfer, and biotransformation of drugs in different therapeutic areas.

Despite these efforts, there are still several issues hindering the study of new ARV drugs in this population during the early phases of drug development (for example, nonuniformity in design and lack of standard methodology in conducting initial safety and PK studies in pregnant and lactating women). Legislation and regulations are in place to both incentivize and mandate trials in the pediatric population; however, these mandates are not in place for trials in pregnant women. A number of potential solutions have been identified to address the need for data in the absence of legislative requirements, but no consensus on standard procedures has been reached. For this reason, the International Maternal, Pediatric, Adolescent AIDS Clinical Trials (IMPAACT) network and the WHO-convened Pediatric Antiretrovirals Working Group (PAWG) collaboratively organized a workshop in June 2019 to define key standards for generation of pharmacology data for ARVs to be used among pregnant and lactating women for HIV prevention and treatment. The IMPAACT/WHO workshop was designed to reach consensus on optimal methods for the design, analysis, and interpretation of pharmacology studies in PLWHIV and associated medical conditions. The objective of the meeting was to expand on existing principles outlined in the Pregnant and Breastfeeding Women module of the WHO “Toolkit for research and development of pediatric antiretroviral drugs and formulations”^25^ and reach a consensus on the optimal design and analysis of ARV pharmacology studies in PLWHIV, review the ARV product pipeline, including long-acting and novel delivery platforms, identify key scientific gaps for use in LMIC, and identify immediate opportunities to undertake optimal studies and rapidly close the knowledge gaps.

## METHODOLOGY

The workshop entitled “Approaches to Optimize and Accelerate Pharmacokinetic Studies in Pregnant and Lactating Women” was held on June 13–14, 2019 in Washington DC, United States. Over forty experts from the IMPAACT network, as well as regulatory agencies, nongovernmental organizations, members of PHASES and PRGLAC, other researchers working in this arena, stakeholders from high-income and low-income countries, clinical and research experts involved in PK studies and *in silico* modeling of ARVs in pregnant and lactating women, participated in the workshop. The meeting was a 1.5-day workshop, starting with a plenary session to review relevant background and objectives, after which breakout sessions were held to facilitate critical review and innovative thinking on the following key topics: ethics and regulatory principles, study design, and analysis and interpretation of pharmacology studies in pregnant and lactating women. Breakout sessions were prepared prior to the meeting by the organizing committee defining objectives and subjects to be addressed. Feedback and plenary discussions enabled the group to reach consensus on key principles and follow-up actions.

## CURRENT PRACTICE

Pharmacokinetic studies involving PLWHIV have historically been performed in the postmarketing phase using an opportunistic approach and is usually performed by independent (academic) research groups. Studies by the clinical trial networks in the United States (IMPAACT P1026S, NCT00042289) and Europe (Pharmacokinetics of Antiretroviral Agents in HIV-infected Pregnant Women (PANNA), NCT00825929) have been the largest and most robust studies of ARVs in pregnant women conducted over the past 15 years. The P1026s and PANNA studies are similar in design, which facilitates the conduct of joint data analyses and presentations as needed. Pharmaceutical companies may collaborate with these networks to perform pregnancy-related studies.

The P1026s (which opened in 2003) and PANNA studies (opened in 2008) are both open-label, parallel-group, multicenter studies utilizing an opportunistic design. Pregnant women are enrolled who are receiving a specific antiretroviral drug as part of their clinical care. ARVs targeted for these trials are those approved for use in nonpregnant adults, but with limited or no safety and PK data in pregnancy. Women are studied longitudinally during pregnancy in the second trimester, third trimester, and postpartum with intensive PK sampling at each timepoint (Table 1). Secondary analyses of PK data generated through P1026S and/or PANNA can then be evaluated with pharmacokinetic modeling methods, including physiologically-based pharmacokinetic (PBPK) and population pharmacokinetic modeling. These modeling approaches can provide generalizable knowledge about drug disposition during pregnancy and postpartum that can inform clinical practice.

Other study designs that have been used to study ARV perinatal pharmacology, also opportunistic in nature, collect sparse samples from women on ARVs during pregnancy and apply population PK modeling to predict the pregnancy effect on PK parameters and to identify covariates that explain intersubject variability, and/or find optimal dosing regimens in pregnancy by simulations. Samples of cord blood and maternal plasma at delivery are typically collected for drug concentration measurements. These data determine the ratio of drug concentrations in cord blood/maternal plasma in order to estimate placental transfer. Recent arms in P1026S have also included infant sampling to assess the infant washout kinetics of transplacentally acquired drug, providing an initial description of drug elimination in the newborn which may be leveraged to support subsequent studies of ARV safety and pharmacology in neonates. Lactation studies are mainly opportunistic studies using a sparse sampling strategy. They focus on the transfer of the ARV into breastmilk, fewer also assessing infant exposure. As these studies are typically performed in LMIC settings where breastfeeding is the recommended practice, there are available breastmilk PK data on the most commonly used ARV drugs in LMICs, but data are lacking on ARVs that are used more frequently in high-income countries.

## DEFINING KEY PRINCIPLES FOR ACCELERATION OF PHARMACOLOGY STUDIES IN PREGNANT & LACTATING WOMEN WITH HIV

### Ethics and regulatory principles

The best way to accelerate availability of PK data of ARVs in pregnancy and lactation is to perform pregnancy studies during drug development, prior to drug approval and marketing. Relatively small PK studies will not provide a full safety or efficacy profile of the drug during pregnancy or lactation, but this information can help clinicians and patients make informed decisions on drug selection and dosing during these important periods. Model-based approaches such as PBPK modeling should be applied to leverage available preclinical and clinical data in order to inform study design and advance protocol development.

#### Pregnancy PK studies during clinical development (preapproval) of a new drug.

The ethical and regulatory principles governing the conduct of these studies in the United States are defined by the Federal Policy for the Protection of Human Subjects (the “Common Rule”). Additionally, the US Department for Health and Human Services (DHHS) regulations include Subpart B—Additional Protections for Pregnant Women, Human Fetuses, and Neonates Involved in Research. While these regulations apply to research conducted or supported by DHHS, they are applied broadly by the FDA. One of the 10 requirements for conducting research in pregnant women specified in the Subpart B is availability of data from preclinical and clinical studies, including studies on pregnant animals and nonpregnant women.^26^ This requirement is intended to facilitate proper assessment of potential risks to pregnant women and fetuses. The working group defined the following prerequisites and needs to accelerate the conduct of PK studies in pregnancy:

1. *Conduct reproductive toxicity studies during early preclinical stages of drug development if there is a likelihood that the target population will include pregnant women*. Additionally, it is important to improve preclinical models of reproductive toxicity to better recapitulate the pregnancy microenvironment and prenatal development.
2. *Institutional Review Boards’ (IRBs’) targeted training* on proper assessment of risk–benefit based on preclinical data will enhance their capacity to evaluate study protocols involving pregnant and lactating women.
3. *Remove regulatory barriers to pregnant women’s participation in clinical research*. Importantly, this is one of the key recommendations in the PRGLAC report. One approach is to modify Subpart B of the DHHS regulations to add in the option of “minor increase over minimal risk,” similar to Subpart D for children.^24^ This will be important where IRBs perceive greater than minimal risk and no direct benefit to an individual pregnant woman, but believe a proposed study is likely to yield generalizable knowledge. Endorsement of such changes by the FDA, EMA, WHO, and other agencies will further strengthen IRBs’ confidence in supporting early-stage pregnancy studies. A driver for industry to perform PK studies in pregnancy could be a minimum number of pregnant women to be included in clinical trials to get marketing approval of a drug, if the drug is likely to be used in pregnancy. A potential unintended consequence of this measure could be a delay in marketing of a new drug. Therefore, this measure should be considered along with other regulatory options.
4. *Allow women enrolled in phase II/III trials to decide if they would like to continue an investigational drug if they become pregnant on study, having fully considered the known and unknown risks and benefits*. If dose-finding studies in nonpregnant participants have been completed and the optimal dosing has been established, this will provide an opportunity to collect PK data to assess the influence of pregnancy on drug exposure. This must be accompanied by close monitoring to collect data on safety and pregnancy outcomes. For studies with the new long-acting ARVs in development, exposure can continue for up to a year after an injection. An option would be that every phase III clinical trial including female participants of childbearing potential should have a substudy in place to collect PK samples in women who get pregnant during the trial, provided that preclinical toxicology studies are completed and do not show alarming safety results. These studies should include collection of ante-natal and postnatal maternal, cord, and infant blood samples (as exposure to the drug over the entire pregnancy and early postpartum period is unavoidable) and safety data. A similar approach in a phase I trial should only be considered if there is no alternative therapy and the potential benefit of continued treatment outweighs the risks. These include the risk of continued fetal drug exposure, of withdrawing the investigational drug from the mother and of fetal exposure to a second drug if the mother is switched to an alternative drug.
5. *Consider single-dose studies with newly developed ARVs on top of optimized background therapy to get insight into the effect of pregnancy on PK, as innovative design*. These types of studies may be more difficult to justify as there is no clear benefit for the individual pregnant woman. However, if harm is suggested to be negligible, this approach may help generate important new information that is likely to benefit future pregnant women. An important consideration will be the drug’s established safety profile as this will determine if the study would be considered a minimal risk or “minor increase over minimal risk” study.

#### Clinical lactation studies during clinical development of a new drug if there is anticipated use by women of reproductive age.

More than 80% of mothers in all settings breastfeed and the duration of (exclusive and nonexclusive) breastfeeding is reportedly highest in LMIC.^27^ Hence, understanding the extent of infant exposure to maternal drugs used for chronic or acute medical conditions is crucial to properly assess potential consequences: theoretical concerns for toxicity from high levels of exposure, prophylaxis with adequate exposure, or resistance development from subtherapeutic ARV exposure among infants who acquire HIV infection. Importantly, there is no need for extra preclinical data to justify a clinical lactation study. Existing ethics and regulatory frameworks support the conduct of PK studies in lactating women receiving a drug prescribed as part of standard clinical care. Examples exist of opportunistic PK studies that have significantly expanded our understanding of the clinical pharmacology of drugs used by lactating women. To further advance this field and obtain these data at earlier stages of drug development, the following recommendations are proposed:

1. *Include phase I clinical lactation studies as part of phase III trials:* Based on FDA guidance, breastfeeding will need to be temporarily interrupted (a woman may pump and discard her milk and feed her infant stored breastmilk) for the duration of the study to prevent infant exposure to an investigational drug.^28^ To get some insight into breastmilk transfer under this recommendation, milk could be pumped just after delivery (before the first breastfeeding session) in women who received the study drug during pregnancy in a phase III trial. An alternative approach is to include lactating women in phase III trials, in a setting where breastfeeding is recommended. Significantly, the FDA recommends non-compartmental and/or compartmental modeling approaches in the analysis of lactation PK data. PBPK modeling is especially promising to predict infant exposure where only breastmilk drug concentration data are available.^29^ An important consideration in using a PBPK approach in lactation studies is the availability of reliable drug and system parameters for both the maternal model and the “infant submodel” to adequately describe drug absorption, distribution, metabolism, and elimination, including the milk-to-plasma ratio. Predictions from such models may serve as a basis for IRBs to evaluate the safety of prospective studies to evaluate infant exposure in a follow-up clinical lactation study. PBPK models may also be a useful approach in evaluating potential changes in drug exposure during the pregnancy-lactation transition period.
2. *Encourage clinical lactation studies in LMIC:* The contribution to infant health and the advice of breastfeeding in these countries compared with higher income countries presents an opportunity to impact public health and accelerate availability of lactation PK data. Reducing the time lag in new drug availability in these settings and making life-saving medications available as soon as possible after approval will help build trust and facilitate early clinical lactation studies which can be conducted as opportunistic studies. Training and retraining of IRBs in LMIC will help facilitate these studies as the regulatory and ethical landscapes change. Endorsement statements of relevant agencies and organizations, including the WHO, FDA, and EMA, may help to get ethical approval and reach consensus on informed consent process, as the infant is a subject in the trial, with potentially a need for both maternal and paternal informed consent.

### Optimal design and analysis of PK studies during pregnancy

**Figure 1:**
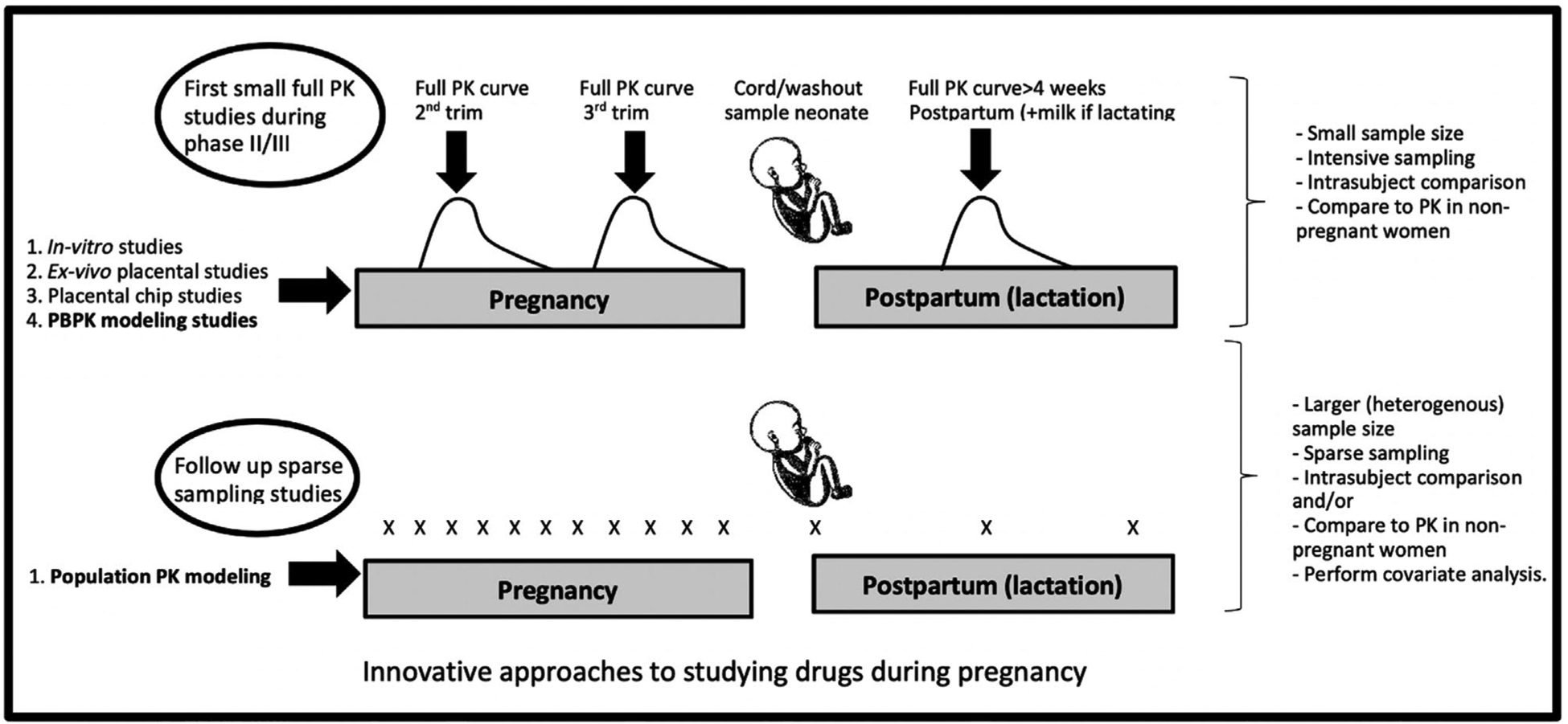
Innovative approaches to studying drugs during pregnancy and lactation. PBPK, physiologically-based pharmacokinetic; PK, pharmacokinetic.

The principles presented here, including the numbers of participants required, are focused on the assessment of PK end points (not safety or efficacy) during pregnancy and lactation. Studies should be designed based upon best available integrated knowledge, including from model-based approaches, such as PBPK modeling. See Figure 1 for a summary of these principles.

#### Design of pregnancy PK studies

##### Conduct an initial PK study to confirm if pregnancy alters a drug’s disposition.

This option is possible in the context of ongoing phase III trials when enrolled participants become pregnant and re-consent to remain on the study drug. To optimize and accelerate the collection of PK data, there is a need for guidance to ensure collection of essential demographic data and use of an appropriate sampling schedule, facilitating analysis of PK data pooled across different studies. These analyses can then be followed up with larger sparse sampling PK studies in pregnant women to explore the effects of clinical and biologic factors on ARV PK and develop optimal dosing regimens using population PK modeling and interventional studies to assess optimal dosage.

##### Choice of study and reference groups.

It is essential that the primary study population for a pregnancy PK study should adequately represent the population where the drug will be used, including in age, body weight, diet, and ethnicity. Consideration should be given to the need to evaluate the potential additional influence of diet, pharmacogenetics, drug–drug interactions (DDIs), comorbidities, and obesity on drug PK in subgroups of pregnant women. For drugs used to treat chronic medical conditions, an intrasubject comparison is the ideal approach for pregnancy PK studies, specifically, assessing PK in the same women during pregnancy and postpartum, with the optimal window for postpartum sampling at least 4 weeks or later after delivery (although it was also agreed that more data defining the optimal PK sampling time postpartum are needed). Using intrasubject comparison helps to minimize interindividual variability. When intrasubject comparisons are not feasible, comparison with pharmacokinetic data from nonpregnant women is a reasonable alternative, although such information can be difficult to obtain because male and female data are often presented together in publications and regulatory reviews. Wider public availability of individual data from clinical studies would enable a better comparison and homogeneity in reporting results. Facilitation of access to sex-disaggregated PK data from phase I/II studies by the industry sponsors and the FDA is recommended to further enable analysis of these studies.

##### Sampling schedule.

The sampling schedule to be used depends on the characteristics and prior knowledge about the disposition of the specific drug being studied. In addition to the timing of sample collection, the number of subjects and the number of samples per subject determine the reliability of parameter estimation in population pharmacokinetic analysis. It is impossible to adopt a standardized sampling strategy for all drugs. These are important considerations when samples are collected to optimize better understanding of PK parameters of interest and practicalities of sample collection. The recommendations in Table 2 provide general guidelines for sampling strategies for pregnancy PK studies. For sparse PK sampling at steady state, it is important to collect the time of last dose and sampling time, usually two samples approximately a half-life apart. Where a single-dose study is proposed, samples should be collected beyond the usual dosing interval to describe a full PK profile. The sampling strategy required for long-acting agents is different, so that what constitutes intensive PK sampling for a long-acting drug will necessarily include collecting sparse samples over a period of weeks or months. Drug and formulation characteristics will determine the optimal sampling strategy. When no data are available to provide a starting point, pregnancy PBPK models may accelerate protocol development and implementation. The typical sample matrix is plasma, but other matrices such as dried blood spots, cells, urine, and possibly hair may be informative. Some alternative biomatrices, e.g., dried blood spots, require minimal processing and avoid shipping restrictions associated with shipment of biohazard samples. For highly protein-bound drugs (> 85%), an effort should be made to determine both total and unbound plasma concentrations.

##### Assessing fetal exposure to maternal drugs.

This requires collection of maternal, cord, and newborn blood samples at delivery. Infant washout samples should be collected up to eight times the adult half-life of a drug after birth, with a minimum of two and maximum of four samples per infant. A short sampling schedule can be proposed for drugs expected to have a short neonatal half-life. Cord-to-maternal blood ratio will also help to estimate the extent to which the study drug accumulates on the fetal side of the placenta, and plasma drug concentration in the cord blood is an acceptable surrogate for drug exposure in late third trimester (when samples in the neonate are not available). Drug metabolism in the newborn can be significantly different from that of adults. Assessment of infant washout elimination is important to assess the period of infant exposure to the drug, informing evaluation of possible infant toxicity and facilitating development of dosing regimens for prophylaxis and early treatment of neonates.

A number of studies have demonstrated the potential role of PBPK modeling in evaluating prenatal drug exposure.^30–32^ Inadequate data on drug disposition in human placenta and fetus can significantly limit the application of these models. However, the application of rigorously validated models will expand our capacity to study drug PK during pregnancy to the prenatal period.

#### Design of lactation PK studies.

A postpartum lactation PK study should be designed to determine the amount of drug ingested by the breastfeeding infant and the extent to which it becomes available in the infant’s systemic circulation. As with pregnancy PK studies, direct observation of maternal drug dose that precedes lactation PK sampling or other measures of adherence will help. Determination of drug protein binding in breastmilk is not thought to be a priority in optimizing and accelerating lactation PK studies.

##### Study and reference groups.

The general considerations are similar to those highlighted for pregnancy PK studies, including the need to have participants that adequately represent the target population and subgroups to evaluate the additional influence of other factors. Importantly, postpartum women who are not breastfeeding can be enrolled in single-dose or short-course dosing lactation studies. This can be done in women living with HIV not intending to breastfeed, or in breastfeeding women without HIV as a single-dose study, to generate maternal plasma/breastmilk ratios (immediately after delivery, before weaning). These studies could be performed in parallel with phase III clinical trials, with the specific enrolment of lactating mothers and their infants into a lactation safety and PK substudy. Furthermore, if a woman enrolled in a phase III trial becomes pregnant and is retained in the study, once she delivers, lactation PK evaluations in both mother and breastfed infant should be undertaken. The information from these studies could help inform PBPK modeling to evaluate infant exposure.

##### Maternal sampling.

Maternal plasma, breastmilk, and infant plasma should be collected in lactation PK studies. Since breastmilk drug concentrations often lag behind plasma concentrations, resulting in changes in the milk-to-maternal plasma ratio, it is important to beware of interpreting ratios from single timepoint data. Breastmilk samples should be collected by manual expression in a clean container, and drugs should be measured in whole milk, without removing the lipid fraction prior to extraction. Dried milk spot assays are under evaluation and may prove useful in low-resource settings, but still require additional assay validation and correlation studies.^33–36^ Intensive PK breastmilk sampling (with as many as seven sampling points over a 24-hour dosing interval) with matched plasma sampling collected from 10–20 participants throughout the dosing interval is recommended as it allows a better assessment of breastmilk penetration of study drugs.^34^ While a rigorous assessment of drug concentration in different fractions of breastmilk (e.g., foremilk vs. hindmilk) may provide some useful information, it was considered unimportant in assessing infant exposure. Complete 24-hour expression of milk from both breasts was thought to be impractical and unnecessary and may be viewed as inappropriate in some settings.

##### Infant sampling.

The most objective index of infant exposure to maternal drug is drug concentration in infant plasma. Repeated dosing of the infant through frequent breastfeeding often results in limited changes in infant plasma concentration during maternal dosing interval. Therefore, it is generally sufficient to collect 2–3 blood samples during a single dosing interval. Data describing the specific time of feeds and their duration are often difficult to collect and may be unnecessary. Other matrices to consider in assessing infant exposure are urine and intracellular drug concentrations. The use of dried blood spot and dried breastmilk spot as micro sampling techniques are acceptable if validated assays are available as reported for some non-nucleoside reverse-transcriptase inhibitors,^36,37^ nucleoside reverse-transcriptase inhibitors,^38,39^ and protease inhibitors.^35^

##### Estimation of infant dosing.

The preferred metric to describe infant dosing is the relative infant dose estimated from drug dose from maternal breastmilk (milk drug concentration*milk volume, where the average milk volume ingested is set to 150 mL/kg/day) and the usual weight-adjusted therapeutic dose in infants (where available) or adults. However, since the bioavailability of drugs in breastmilk is generally different from other standard vehicles, the relative infant dose should be interpreted with caution.

### Interpretation of PK study results

#### Interpretation of data from pregnancy PK studies.

**Figure 2:**
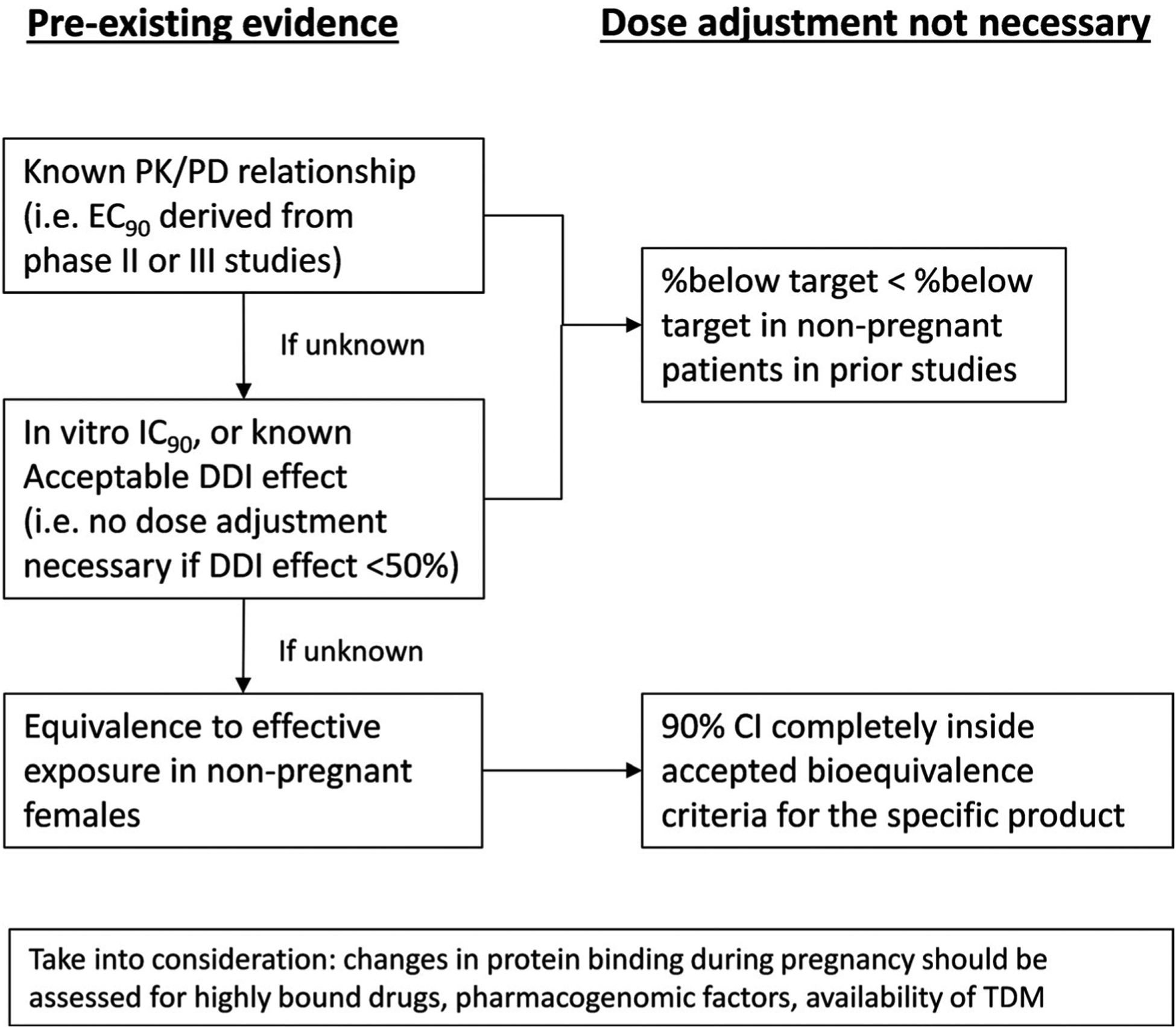
Algorithm for interpretation of PK studies in pregnancy. CI, confidence interval; DDI, drug–drug-interaction; EC_90_, 90% maximal effective concentration; IC_90_, drug concentration resulting in 90% inhibition of viral replication (*in vitro*); PK/PD, pharmacokinetic/pharmacodynamic; TDM, therapeutic drug monitoring.

In line with FDA recommendations, PK parameters of the parent drug and its active metabolites (where applicable) should be estimated from total and unbound plasma concentration data.^40^ The target value of the primary parameter (e.g., area under the concentration-time curve (AUC) over the dosing interval, C_trough_, concentration at the end of the dosing interval) in a pregnancy or lactation PK study should be based on the strongest preexisting evidence. The strongest evidence for establishing target ranges for PK parameters is considered to be a robust relationship between PK and efficacy (e.g., PD), providing a widely accepted target for therapeutic drug monitoring purposes. If no target based on a strong PK/PD relationship is available for a drug, the next lower level of preexisting evidence would be considering other PK/PD properties and characteristics of the drug, such as protein-binding adjusted inhibitory or effective concentrations (IC_90_, IC_50_, EC_95_, etc.) or known acceptable magnitude of DDIs in nonpregnant adults (Figure 2). For example, if according to the product label a DDI of a magnitude of 50% reduction of AUC is acceptable and does not lead to dose adjustments or contraindications, such a reduction would be acceptable in pregnancy. In these cases, the percentage of pregnant women below the relevant PK target should not be more than the percentage of nonpregnant participants below target in prior DDI studies.

When no data are available to inform target exposures, the next best approach would be to try to match plasma drug exposures during pregnancy (AUC_0-tau_, area under the concentration-time curve over the dosing interval) to those exposures shown to be effective in nonpregnant females. The bioequivalence approach may be used to assess the differences in AUC (geometric mean ratio) and 90% confidence interval (CI) during pregnancy vs. postpartum. The 90% CI should typically fall between 80 and 125%, however, a wider range (70–143%) can be considered for potent drugs with a large safety margin.^41^ Pharmacokinetics may also be altered in the immediate postpartum period. Postpartum PK data should therefore be compared with historical nonpregnant PK data (preferably in women) prior to drawing conclusions about altered exposure during pregnancy.

For highly protein-bound drugs, changes in protein binding during pregnancy should also be factored into these determinations of target exposures, as total drug concentrations may be affected in pregnancy, whereas unbound drug concentrations may remain unchanged.

If pharmacogenomics plays a substantial role in drug exposure, this should be taken into account when deciding on the acceptability of decreased drug concentrations in pregnancy, as pregnancy may modify the effect. Furthermore, in a setting where therapeutic drug monitoring is available, dosing can be based on therapeutic drug monitoring results, which can support individualized dosing in pregnancy.

See Figure 2 for a summary of these recommendations.

#### Interpretation of Data from lactation PK studies.

Many of the same considerations discussed for interpreting studies in pregnant women also apply to interpreting lactation studies. Again, the key PK parameter to study will depend on what is already known about the drug product. In lactation studies, the key PK parameter may also depend on the sampling strategy and which PK parameters (e.g., AUC, concentration ratios, and estimated relative infant dose) are available for interpretation. Interpretation of breastmilk concentrations may be important for considering viral suppression and the potential for viral resistance in this compartment in the mother but will likely be primarily important for considering the infant’s drug exposure. When evaluating data from lactation studies of ARVs, it is important to remember that in general, drug concentrations are low in breastmilk,^42^ and that higher drug exposures in breastmilk may not necessarily result in significant neonatal/infant exposure, but could achieve exposures effective in preventing HIV acquisition. Somewhat lower exposures may be within the therapeutic range (if known) for infant prophylaxis against acquisition of HIV. When infant exposure is lower than therapeutic exposures but still clinically relevant, the infant may be at risk for selecting resistance mutations in cases of vertical transmission. However, this overall risk is low since the number of infants who acquire HIV infection through breastfeeding while the mother is adherent and virologically suppressed on ART is small. The risk/benefit ratio in this scenario would need to be carefully evaluated. With very low to unmeasurable concentrations, no clinically significant effects would be expected in the infant. When considering ARV exposure through breastfeeding in the infant, a multitude of factors may also need to be considered, such as pharmacogenetics, DDIs, maternal adherence, prematurity, ontogeny of drug absorption, distribution, and metabolism/elimination pathways in the infant, and the interplay of all these factors with the gut microbiome.

## ARV PIPELINE AND OPPORTUNITIES FOR ACCELERATION OF PREGNANCY AND LACTATION STUDIES

The ARV drug development process remains active, with many drugs currently in phases I, II, and III stages of development (Table 3). With improved drug development, newer drugs are less toxic, longer lasting, and more efficacious.^43^

Two recently marketed ARVs—bictegravir and doravirine—will be investigated in pregnant women within IMPAACT 2026 and PANNA. Furthermore, a phase Ib study of bictegravir in pregnancy (NCT03960645) is being performed by Gilead Sciences.^44^ For ibalizumab, a recently marketed monoclonal antibody for treatment of multidrug resistant virus, no studies in pregnancy are planned. For this monoclonal antibody no reproductive toxicology studies have been performed, but monoclonal antibodies are likely to cross the placenta and reach the fetus. The likelihood of including pregnant women on ibalizumab in opportunistic design studies as IMPAACT P2026 and PANNA is very low, because the number of pregnant patients with multidrug resistant virus in need of these drugs is likely to be very limited. For ibalizumab, we recommend individual case reports to be collected and, if possible, PK samples be obtained for any woman receiving the product who becomes pregnant.

HPTN 084 (A Phase 3 Double Blind Safety and Efficacy Study of Long-Acting Injectable Cabotegravir Compared to Daily Oral TDF/FTC for Pre-Exposure Prophylaxis in HIV-Uninfected Women)^45^ will not include pregnant women, or women who want to become pregnant. In the current study design, cabotegravir injections will stop if a woman becomes pregnant, but the women will return for regular visits to store plasma and dried blood spot, as well as safety follow-up, and peripartum and lactation PK sampling will be performed as part of IMPAACT P2026. MOCHA (More Options for CHildren and Adolescents, IMPAACT 2017) and BREATHER PLUS will study cabotegravir in children and/or adolescents. In these studies, participants will stop injections when pregnant, but PK samples will be collected when a woman becomes pregnant.

Dapivirine, an NNRTI to be used for preexposure prophylaxis as 25 mg dapivirine vaginal ring, is expected to be used in lactating women as breastfeeding is supported in countries with high HIV incidence. Therefore, breastmilk and maternal concentrations during breastfeeding were assessed in a short course PK study in 16 (healthy) women. In both breastmilk and maternal plasma, low dapivirine levels were detectable. Terminal half-life of 39 hours in milk and 35.2 hours in plasma were reported resulting in an estimated infant exposure of 65 ng/kg/day through breastmilk.^46^ A study on safety and drug detection of dapivirine vaginal ring in breastfeeding mother–infant pairs (NCT04140266) is planned, and a study of the safety of dapivirine vaginal ring in pregnancy (NCT03965923) is recruiting. The latter study evaluates the maternal and infant safety of two preexposure prophylaxis methods (dapivirine vaginal ring and daily oral Truvada (emtricitabine and tenofovir disoproxil fumarate)) in 750 HIV-uninfected pregnant women and their infants. Safety of mother and child are the primary objectives, but also include dapivirine levels in the mother during pregnancy and the infant after delivery.

**Figure 3:**
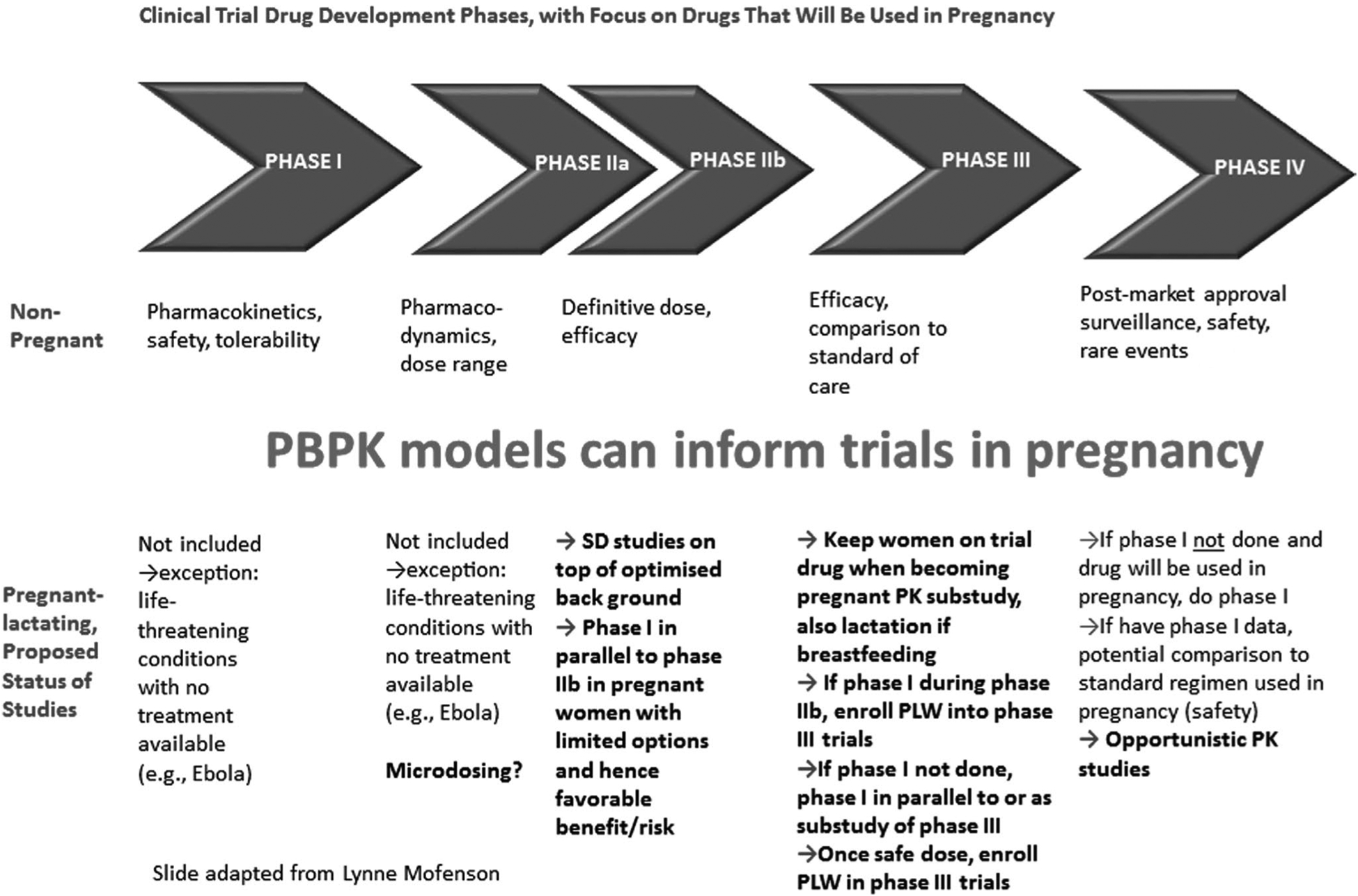
Summary principles for optimal PK studies of new ARVs in pregnant and lactating women. PBPK, physiologically-based pharmacokinetic; PLW, pregnant and lactating women; PK, pharmacokinetics; SD, single dose.

For all other new ARVs, no information on planned pregnancy or lactation studies are available, to our knowledge. It is imperative that this population, PLWHIV, be included in planned studies of new agents as described in Figure 3 where optimal timing and steps are proposed. This is especially important for long-acting ARVs: a PK in pregnancy substudy should be part of all studies with participants of reproductive age acknowledging the large potential for pregnancy while on study. We strongly advocate that all phase III study protocols for long-acting drugs (both for treatment and preexposure prophylaxis) contain such a substudy. This also means that the necessary preclinical reproductive toxicology studies should be completed prior to phase III. For all compounds in phase II and III, there is an opportunity for industry to consider keeping pregnant women in the study and perform a PK substudy in pregnancy and lactation and to include pregnant and lactating women in phase III studies. The dapivirine vaginal ring is a great example to show that this is possible.

## CONCLUSION

Several examples demonstrate that as long as pregnant women are excluded from drug development programs prior to regulatory approval, they remain at high risk to receive potentially inadequate treatment until postmarketing pregnancy PK and safety data become available. The consensus of the workshop was that recruitment of pregnant and lactating women into clinical trials must be encouraged. The potential for pregnancy should not automatically exclude a woman from participating in a clinical drug trial. Pregnant and lactating women should be eligible for all phase III ARV trials, and some phase IIb clinical trials (after the necessary preclinical reproductive toxicology studies are completed), unless there is a compelling reason for exclusion. In addition, pharmaceutical companies should be encouraged to include pregnant women in early-phase clinical trials, as these data are required to adequately inform treatment and dosing decisions in healthy and pregnant women living with HIV or other chronic medical conditions. Interdisciplinary collaboration is needed to generate data sets and integrate these data to establish population pharmacokinetic models and verify PBPK models, as dedicated pharmacometric analysis can provide invaluable insights into rational drug use during pregnancy and lactation. While ARV pharmacology is often ahead of other fields in terms of pregnancy and lactation PK data, increasing the use of *in vitro* placental studies and PBPK modeling to elucidate pregnancy-associated changes and factors influencing breastmilk transfer and fetal and infant exposure could improve drug dosing and use during pregnancy and lactation in other therapeutic areas. Finally, clinicians, pharmacologists, policymakers, community members, advocacy groups, researchers, and ethics committees should continue to collaborate in pregnancy-related research, and take necessary steps to facilitate inclusion of pregnant women in clinical research to help answer important questions about the effects of medication use during pregnancy and lactation and the ways in which pregnancy alters PK of drugs.^16^

## Figures and Tables

**Table 1:** Summary of study designs of IMPAACT P1026S and PANNA protocols used to study select ARVs in pregnant and lactating women living with HIV

	IMPAACT P1026S	PANNA
Countries	USA/Thailand/South America/Sub-Sahara Africa	Europe
Timing	2^nd^, 3^rd^ trimester of pregnancy, postpartum PK curves (intensive sampling)	3^rd^ trimester of pregnancy, postpartum PK curves (intensive sampling)
Number of participants	25 women 3^rd^, ≥ 12 women 2^nd^ trimester	≥ 16 women 3^rd^ trimester
Statistics	Intrasubject comparison	Intrasubject comparison
Timing analysis	PK assessment real time, stopping criterion if a predefined number of women fall below the target drug exposure	Batch analysis
PK method	Noncompartmental analysis (NCA)	NCA
Comparison statistics	Comparison with 10th percentile for the nonpregnant population (< 20% below this target is acceptable)	Bioequivalence approach for analysis of PK data
Delivery PK	Cord blood/ maternal blood at delivery	Cord blood/ maternal blood at delivery
Infant PK	Infant washout samples	—

ARVs, antiretrovirals; PK, pharmacokinetics.

**Table 2:** Summary of recommendations on sampling strategies for pregnancy PK studies

Type of sampling	Method of analysis	Number of sampling points and participants^a^	Trimester to sample
Intensive PK samples	Noncompartmental analysis (NCA)	Usually 7–12 samples over one dosing interval at steady state from 12–24 participants	Preferably first, second, and third; or second and third; or early third (28–32 weeks of gestation) plus sparse sampling at early visits.
Sparse samples	Nonlinear mixed effects (NLME) modeling	Randomly assign participants to sampling windows; or Patients randomly contribute two or more samples to cover dosing interval; or Most patients contribute one sample at a specified timepoint.	

PK, pharmacokinetics.

a For intensive PK studies, the number of participants should be sufficient to detect changes in the primary PK parameters that warrant dosage adjustments.

**Table 3:** ARV pipeline

Drug name	Class	Development phase	Dosing regimen	Treatment indication	PK studies in pregnancy/ lactating	PK studies in pregnancy planned
Bictegravir	INSTI	Marketed	50 mg oral q.d. (combination with TAF/FTC)	HIV treatment		Gilead sponsored Phase lb study recruiting (NCT03960645); IMPAACT P2026; PANNA
Doravirine	NNRTI	Marketed	q.d. dosing oral, nanoformulation LA?	HIV treatment		IMPAACT P2026; PANNA
Ibalizumab-uiyk	MAB	Marketed	IV loading dose of 2,000 mg; maintenance dose of 800 mg every 2 weeks	HIV Treatment Multidrug Resistant HIV-1		Unknown
Dapivirine	NNRTI	Phase III	vaginal ring, 1/month	PrEP	25 mg dapivirine ring, day 14 PK in 16 (healthy) lactating women.	Study in pregnancy recruiting: 750 women (dapivirine vs. Truvada) + PK mother and child (NCT03965923)
Cabotegravir	INSTI LA	Phase III completed	LA (IM), 1/month (or longer)	HIV trt and PrEP		HPTN 084, Phase III, NCT03164564 (recruiting) if particpant becomes pregnant: stop injections; regular visits, store plasma and DBS. IMPAACT P2026 and PANNA will add to list.
Fostemsavir	Attachment inhibitors MDR HIV	Phase III completed	Oral 600 mg b.i.d.	HIV Treatment Multidrug Resistant HIV-1		Unkown
UB-421	CD4 attachment inhibitor	Phase III	IV, 1/week or /2 weeks	HIV treatment		Unkown; NCT04041362 phase II study seems not to exclude pregnant women.
Islatravir MK-8591 (EFdA)	NRTTI	Phase III	Oral 1 tablet/day or week; s.c. implants 2/year?	HIV trt and PrEP		Unkown
Albuvirtide	Fusion inhibitor	Phase II/III	LA, per 2 weeks or every 4 weeks; IV infusion	HIV treatment		Unkown
PRO 140/ Leronlimab	CCR5 agonist MAB	Phase II/III	LA, s.c. inj weekly	HIV treatment		Unkown
Elsulfavirine/ Elpida (VM1500; VM1500A)	NNRTI	Phase II/III^a^	LA?, oral 20 mg once daily? Injection??	HIV trt and PrEP		Unkown
ABX464 (Abivax)	Rev inhibitor	Phase II/III	oral, once daily	HIV treatment		Unkown
VRC01/VRC01LS	therapeutic vaccine, bNAb	Phase II	LA, IV, or s.c. 1/3weeks	HIV trt and PrEP		Unkown
GS-9131 - prodrug for GS-9148	NRTI (works against NRTI resistant virus)	Phase II	Oral, once daily	HIV treatment		Unkown
GSK3640254	maturation Inhibitor	Phase IIa	Oral, once daily	HIV treatment		Unkown
GS-6207	Capsid inhibitor	Phase I/IIA	LA, s.c. injection 1/3 months	HIV treatment		Unkown
GS-CA1	Capsid inhibitor	Preclinical	LA, s.c. ?	HIV treatment		Unkown
Combinectin (GSK3732394)	Adnectins and fusion inhibitor peptide	Preclinical	LA, s.c. weekly dose	HIV treatment		Unkown

bNAb, Broadly neutralizing antibodies; CCR5, CC chemokine receptor 5; DBS, dried blood spot; FTC: emtricitabine; IM, intramuscular; inj, injection; INSTI, integrase strand transfer inhibitor; IV, intravenous; LA, long acting; MAB, monoclonal antibody; MDR, multidrug resistant; NNRTI, non-nucleoside reverse-transcriptase inhibitors; PrEP, preexposure prophylaxis; q.d., once daily; s.c., subcutaneous; TAF, teneofovir alafenamide; trt, treatment.

a Marketing approval Russia 2017.
